# Health behaviours and mobile intervention use in patients recruited from general practitioners’ practices in rural Bavaria

**DOI:** 10.1080/21642850.2024.2444244

**Published:** 2024-12-23

**Authors:** Laura M. König, Constanze Betz, Mirna Al Masri, Tina Bartelmeß

**Affiliations:** a Faculty of Psychology, University of Vienna, Vienna, Austria; b Faculty of Life Sciences: Food, Nutrition and Health, University of Bayreuth, Kulmbach, Germany; c Faculty of Law, Business, and Economics, University of Bayreuth, Bayreuth, Germany

**Keywords:** MHealth, overweight, food environment, physical activity, diet

## Abstract

**Introduction:** Individuals living in rural areas report poorer health outcomes, including obesity, compared to individuals living in urban areas. Amongst others, this is due to restricted access to opportunities for healthy eating and physical activity. Interventions are urgently needed to address this gap. It is yet unclear whether digital interventions are suited for this purpose. The present dataset provides information on adults residing in rural Germany regarding their health status, perceived access to opportunities for healthy eating and physical activity, and digital device ownership and intervention use.

**Materials and methods:** A pen-and-paper survey was conducted in winter 2022/2023 among patients of five general practitioners’ practices in rural Bavaria. Materials and data are openly available for future use.

**Data description:** The dataset contains responses from *N* = 273 individuals (54.9% women, 44.8% men, 0.4% diverse; age *M* = 51.3, *SD* = 16.7; BMI *M* = 29.1, *SD* = 15.9). On average, 30.6 participants failed to respond to any given item (*SD* = 33.0; range 1–136). Eighty-four percent had access to the internet and a computer and 91.4% owned a smartphone, but the majority (58.5%/ 84.2%) had no prior experience with mobile physical activity or dietary interventions, respectively.

**Discussion:** This dataset provides insights into barriers and facilitators to healthy eating and physical activity in rural populations and digital (health) technology use. It provides starting points for behavioural weight management interventions in rural areas.

## Introduction

Despite public awareness and longstanding prevention efforts, obesity rates are steadily increasing across age groups in high income countries (Phelps et al., 2024). Obesity, in turn, is an important risk factor for the development of a range of non-communicable diseases such as heart diseases and so significantly contributes to the global burden of disease and reduced quality of life (Powell-Wiley et al., 2021). People living in rural areas show worse health outcomes compared to people living in urban areas, e.g. regarding the prevalence of obesity (Cohen et al., 2018), which translates into higher prevalences of related diseases such as Diabetes mellitus type 2 and coronary heart disease (O'Connor & Wellenius, 2012).

Evidence indicates that physical inactivity and poor diets, two behaviours associated with obesity, are more prevalent in rural vs urban populations (McCormack et al., 2018; Weaver et al., 2013). Indeed, urban and rural populations are differentially exposed and vulnerable to conditions associated with poorer health outcomes; for instance, in rural areas, fewer opportunities to engage in structured exercise (e.g. at fitness centres) are available, and due to longer travel distances, active commuting is less prevalent (Cleland et al., 2015). Similarly, access to supermarkets and healthy foods is often restricted in rural compared to urban areas (Larson et al., 2009).

Despite the increased burden of obesity in rural populations, research on determinants of health and behavioural interventions addressing obesity is predominantly conducted in urban populations (Pratap et al., 2020). This might be due to researchers at universities having easier access to these populations, and individuals living in urban areas having to travel longer distances to reach study centres, which requires more time (Friedman et al., 2015; Krukowski et al., 2024; Lara Jr et al., 2001). More research is needed in rural populations to better understand the reasons for rural-urban health disparities and to develop targeted interventions to make health promotion more equitable.

Digital interventions including the use of smartphone apps, social media platforms, and wearables, have been proposed as a means to improve access to healthcare in rural areas (Albrecht, 2016). However, evidence is lacking for this claim (Chesser et al., 2016), especially regarding rural populations outside the United States. Indeed, there is first evidence for a digital health divide that also extends to rural populations (Muralidharan et al., 2019). Broadbent access is still more limited in rural vs urban regions in Europe (de Clercq et al., 2023), which might then translate into more general differences in digital literacy, and subsequently uptake of and engagement with digital tools (Yahn, 2023). Accordingly, this study aimed to provide insights into willingness and feasibility to use digital interventions for promoting healthy eating, physical activity, and weight loss in a rural German sample to shed further light on potential disparities in digital health promotion and avenues for more equitable digital interventions (c.f. König et al., 2023; Szinay et al., 2023).

The present data was collected as part of a larger transdisciplinary project on obesity treatment in primary care in rural Germany. ‘Rural’ in this context refers to Upper Franconia, a subdivision of Bavaria in Germany with approx. 1 million inhabitants. Similar to other rural areas in Germany, it has a low population density and aging demographic, with 23.2% of residents aged 65 or older and 47.2% aged 50 or older (Statistik Oberfranken, n.d.). Rural areas in Germany are typically defined by small settlements, limited infrastructure, reliance on personal transportation, and economies based on agriculture, forestry, or small-scale industries. These regions face challenges such as aging populations, youth migration to urban areas, and reduced access to services due to the closure or centralisation of facilities like schools, municipal offices, and grocery stores, which diminishes spatial accessibility (Hernández et al., 2024; Kopfmüller, 2008) as well as higher prevalences of noncommunicable diseases such as diabetes mellitus Type 2 (Wissenschaftliches Institut der AOK, 2019). The present dataset served as a baseline survey of the local population for an intervention study that started recruiting in spring 2023 (study protocol: Haderer et al., 2024). It furthermore informed intervention development by providing information on the perceived constraints and opportunities for healthy nutrition and physical activity in rural areas to address possible inequalities in health due to differences in social and physical environments. In addition, the study was designed to address the following research questions (RQ):

RQ 1: Is there social inequality in the uptake of and engagement with mobile interventions to promote physical activity and healthy eating in a rural population? We focused on the following social inequality indicators, based on the PROGESS-Plus framework (O'Neill et al., 2014): gender, age, years of education, net household income, employment status, migration history.

RQ 2: How do participants prefer to obtain information about nutrition? What reasons can be identified for the use of social media platforms for information about nutrition?

Since the achieved sample size was too small to run the planned analysis for RQ 1 (see Al Masri & König, 2022, for the data analysis plan and the supplementary material for the results as per preregistration) with sufficient statistical power, this data note aims to make the dataset accessible to other research teams for further use, e.g. in meta-analyses, as a comparison sample, or for teaching purposes.

## Materials and methods

The study was preregistered on the Open Science Framework (OSF; https://osf.io/2rngw) prior to collecting data. Study materials (original and translated into English) are available on the OSF (https://osf.io/5vzpy/). The study adhered to the Declaration of Helsinki and received approval from the University of Bayreuth ethics committee (reference number 22-033).

### Sample

We recruited adults aged at least 18 years who spoke sufficient German to read and respond to the questions in the survey. Participants were recruited via five general practitioners’ practices in Upper Franconia between December 2022 and February 2023.^1^ Questionnaires were handed out by the receptionists when patients entered the practice as a means to shorten waiting times; in addition, the GPs approached their patients and asked them whether they were interested in participating. Participants did not receive any compensation for completing the questionnaire. No target sample size was determined since feasibility of the recruitment procedure was unclear.

### Procedure

Pen-and-paper questionnaires were distributed in the waiting areas of five general practitioners’ practices in Upper Franconia, Germany. Participants could either complete the questionnaire while they waited or take the questionnaire home and return it at a later appointment. Participants provided informed consent by ticking a box on the first page of the questionnaire; we did not ask for a signature to preserve their anonymity. The questionnaire contained items covering socio-demographic information, health status, lifestyle, information sources, and technology use.

### Measures

The questionnaire was administered in German. The original version and the English translation (for transparency) are available from the OSF, as is a data dictionary linking the variable names to the questionnaire items: https://osf.io/5vzpy/. Items were derived from consumer surveys and dietary studies, such as the German National Nutrition Survey, and adapted to suit the objectives of this study. If not otherwise specified, items were developed by the authors for descriptive purposes. The questionnaire was assessed for readability and comprehension by a diverse group of 10 individuals of varying ages not otherwise involved in the research project. This evaluation aimed to ensure accessibility for all patients and to verify the accuracy of the estimated completion time.

#### Socio-demographic information

Participants indicated their age (in years), their gender (male/ female/ diverse), their highest school leaving and vocational qualifications (from a list of 7 and 8 options, respectively), whether they held German citizenship (yes/ no), whether they or at least one of their parents immigrated to Germany (yes/ no; assessment of migration history), their monthly net household income (from a list of 11 options), whether they received unemployment benefits (yes/ no/ prefer not to say), and their current employment status (from a list of 6 options).

Based on the highest school-leaving and vocational qualifications, years of education were calculated as the sum of years derived for the two qualifications. The assignments of years to qualifications can be found in Table S1 the supplementary material.

#### Health-related information

We assessed perceived health status (‘How would you rate your health status?’) on a five-point scale from (1) very bad to (5) very good. Intention to lose weight (‘I would like to lose weight in the future’) was assessed on a five-point scale from (1) do not agree at all to (5) fully agree. Participants were further asked to indicate how much they would pay per months for participating in a weight loss programme (‘There are various guided programs that successfully help people to lose weight. What is the maximum amount of money you would pay per month to participate in such a program?’). They also indicated their height and weight. On the last page of the questionnaire, participants were asked to indicate whether they reported height and weight estimates or figures assessed by the GP during their appointment. Female participants were asked to indicate whether they were currently pregnant (yes/ no). Finally, diagnoses of a range of non-communicable diseases (e.g. diabetes mellitus types 1 and 2, chronic lung conditions, high blood pressure) and depression were assessed (all yes/ no).

#### Living situation, including information on nutrition and grocery shopping and leisure time activities

Participants indicated the number of people in their household (‘How many people live permanently in your household, including yourself?’) and the relationship to them.

They were asked whether they usually consumed any main meal (breakfast, lunch, dinner) or snacks at home, outside of home, or not at all. They were also asked to indicate whether they usually consumed these meals alone or with other individuals. Furthermore, they indicated who usually prepared their meals when eating at home (they only for themselves/ they for themselves and for others/ someone else).

Regarding eating- and physical activity-related leisure-time activities, participants indicated how frequently they ate at restaurants or at friends’ or family members’ homes, went for drinks at clubs or bars or at friends’ or family members’ homes, consumed food or drinks at public gatherings. Regarding exercise, they were asked how frequently they exercised in a sports club, a fitness studio, or at home or in nature. Responses were collected on a six-point scale from (1) daily to (6) never.

Participants indicated where they usually buy groceries, selecting up to three from a range of options (e.g. supermarket, farmers market, online). They were also asked to indicate their use of food banks (‘How often do you receive food from the food bank (Tafel) or other food donations?’), ranging from (1) weekly to (4) never, including (5) I do not know.

#### Food environment

To assess perceptions of the food environment, items were adapted from the Perceived Food Environment Questionnaire (Carbonneau et al., 2019; 2017). The first set of questions evaluated the availability of healthy foods (e.g. ‘Healthy/fresh food is easily accessible in my area’), with responses measured on a five-point Likert scale ranging from (1) do not agree at all to (5) agree fully. The second set focused on self-reported travel time from home to the primary food retailer, either by car or on foot (‘How long does it take you to get from your home to the nearest shopping facility on foot?’; ‘How long does it take you to get from your home to the nearest shopping facility by car?’). Response options were ‘less than 10 minutes’, ‘10–20 minutes’, or ‘more than 20 minutes”.

#### Physical activity environment

Participants were asked to indicate the number of opportunities for physical activity in their neighbourhood (1 km around their house), such as playgrounds, outdoor gyms, indoor gyms from (1) none to (4) many (‘How many movement opportunities are there in your neighbourhood (one kilometre radius)? (walking paths, playgrounds, fitness facilities, public fitness equipment, gyms, sports fields, sports clubs)’). They also indicated perceived accessibility of the nearest opportunity for being physically active (‘How easy is it at your place of residence to get to the nearest opportunity for being physically active?’) from (1) very difficult to (5) very easy. Participants furthermore whether they found the opportunities appealing, sufficient, and whether they did not make use of the opportunities due to cost (e.g. ‘I find the offer of opportunities for being physically active in my area appealing’; all on 5-point scales from (1) do not agree at all to (5) fully agree). The development of these items was informed by the recommendations outlined in Eyler et al. (2015).

#### Nutrition information seeking

Participants were asked about the sources which they used to obtain information about food and nutrition and chose up to three from a list of 13 options (or indicated that they did not seek information about food and nutrition). They furthermore indicated with whom they usually talked about nutrition-related topics, including friends or family. Finally, they were asked to select a maximum of three options as to why, if at all, they used social media to obtain information about food and nutrition.

#### Use of digital technology

Following the procedure in König et al. (2018), participants were first asked whether they had access to the internet, owned a computer, a tablet, or a smartphone. If they owned a smartphone, they were asked to indicate the operating system.

#### Use of mobile fitness technology

Participants were then asked to indicate whether they used fitness apps and trackers using the stage model approach introduced by König et al. (2018). This measure builds on the Precaution Adoption Process Model (Weinstein, 1988; Weinstein & Sandman, 1992), which postulates that behaviour change occurs in a series of stages that require qualitatively different intervention approaches. In addition, they indicated how much they would pay for a fitness tracker and a fitness app. Current, and former users of a fitness tracker or app were asked to indicate the model/ app name.

#### Use of mobile dietary applications

Following the same procedure (König et al., 2018), participants indicated their use of nutrition apps and how much they would pay for a nutrition app. Current and former nutrition app users were asked to indicate the name of the app they used.

## Ethics statement

Ethical approval was obtained from the University of Bayreuth ethics committee prior to data collection.

## Data description

Data was analysed using JASP 0.18.3 (JASP Team, 2024).

### Data availability and use

The dataset (in original German as well as an English translation of the responses to the open-ended questions) is available from the project’s OSF page: https://osf.io/5vzpy/. It can be used for non-commercial purposes (CC-BY-NC).

### Data quality

Due to the pen-and-paper format and the staff in the practices checking questionnaires for completeness having been deemed infeasible, the number of missing values on all single-choice variables was relatively high. Missing values ranged from *n* = 1 for German citizenship to *n* = 136 for the amount of money they would spend on a weight loss programme (optional responses or conditional responses excluded), with a mean of 30.6 missing responses (*SD* = 33.0).

### Sample description

A total of *N* = 313 questionnaires were returned, but *n* = 39 participants did not check the box for providing informed consent, and *n* = 1 participants reported to be younger than 18 years of age. This reduced the final number of participants to *N* = 273 (54.9% women, 44.8% men, 0.4% diverse). Mean age is 51.3 years (*SD* = 16.7), and median age 53 years. The vast majority (98.9%) of participants holds German citizenship, and 7.2% have migration history (i.e. they themselves or at least one of their parents immigrated to Germany). Breakdowns of highest school-leaving and vocational qualifications, income and employment status are listed in Table 1. Mean years of education is 13.5 (*SD* = 2.8), and mean BMI is 29.1 (*SD* = 15.9).

**Table 1.** Socio-demographic characteristics of the sample.

Characteristic	%	Median	Interquartile range
**Highest school-leaving qualification**		3	3 (from 2 to 5)
(1) Do not have a school leaving certificate (yet)	0.8		
(2) Elementary school, lower secondary school	31.8		
(3) Intermediate secondary school leaving certificate	27.2		
(4) Completion of polytechnic secondary school	1.5		
(5) Graduation from a (vocational) technical school	21.6		
(6) University entrance qualification (Abitur)	16.8		
(7) Other qualification, namely: ____________________	0.4		
**Highest vocational qualification**		2	2 (from 2 to 4)
(1) Do not have completed vocational training (yet)	7.7		
(2) Vocational training	48.1		
(3) Apprenticeship	15.8		
(4) Master (craftsman)	6.5		
(5) Technical/vocational secondary school	3.9		
(6) Degree from a university of applied science/degree from a university	15.0		
(7) PhD/doctorate/habilitation	1.5		
(8) Other qualification, namely: ____________________	1.5		
**Monthly net household income**		7	3 (from 6 to 9)
(1) less than 150€	1.1		
(2) 150€ to 300€	0.4		
(3) 300€ to 500€	0.4		
(4) 500€ to 1.000€	5.7		
(5) 1.000€ to 1.500€	12.9		
(6) 1.500€ to 2.000€	16.0		
(7) 2.000€ to 2.500€	16.0		
(8) 2.500€ to 3.000€	12.9		
(9) 3.000€ to 5.000€	21.3		
(10) 5.000€ to 10.000€	11.8		
(11) more than 10.000€	1.5		
**Employment status**			
(1) employed (full-time)	45.6		
(2) employed (part-time)	20.4		
(3) seeking employment	3.0		
(4) in training/ retraining/ studying	3.7		
(5) exclusively a homemaker	1.9		
(6) retired	25.6		

Compared to the population of Upper Franconia, the sample contains of slightly more women (population of Upper Franconia: 50.9%; Statistik Oberfranken, n.d.) and substantially fewer participants who do not hold German citizenship (population of Upper Franconia: 8.3%; Statistik Oberfranken, n.d.). The sample also has a higher BMI (population of Upper Franconia: 26.4 kg/m²; Bayerisches Landesamt für Statistik, 2022). Both the sample’s and the Upper Franconian population’s BMI is higher than the average of the population of the state of Bavaria (Bavaria: 25.8; Bayerisches Landesamt für Statistik, 2022). As per Statistik Oberfranken (n.d.), the median age category is 40 to under 60 years, in which the median age of the present sample also falls.

### Health-related technology use

The majority of the sample (84.0%) had access to the internet, owned a computer (84.0%), a tablet (67.2%) and a smartphone (91.4%). The breakdown of fitness apps and trackers and nutrition app use stages is listed in Table 2.

**Table 2.** Fitness apps and tracker and nutrition app use stages, following the behaviour stage model presented in König et al. (2018).

	Stage 1 ‘unengaged’	Stage 2 ‘decided to act’	Stage 3 ‘decided not to act’	Stage 4 ‘acting’	Stage 5 ‘disengaged’
Fitness apps and tracker	46.7%	7.0%	4.8%	30.1%	10.9%
Nutrition apps	73.5%	6.4%	4.3%	5.6%	9.8%

We planned to test for socio-demographic differences in health-related technology use (Al Masri & König, 2022, December 9). Due to the small sample size and unequal group sizes for both fitness tracker and app and nutrition app use and some social inequality indicators (e.g. migration history), we deemed the dataset unfit for these analyses. The analyses are reported as preregistered in the supplementary material.

## Discussion

This survey was designed to provide socio-demographic information for the potential population to be recruited for a multicomponent GP-led intervention which aimed to reduce obesity in rural Bavaria (see Haderer et al., 2024); it also provided information on the potential participants’ barriers to healthy eating and physical activity that was used in intervention development. It furthermore aimed to evaluate current mobile intervention use and nutrition-related information seeking in this population. Due to the relatively small sample size and unequal group sizes, these analyses could not be conducted as planned. It is furthermore important to note that this is a convenience sample that is not representative for the German general population, so conclusions drawn from this dataset might not extended to the whole population. Moreover, since the survey had to be short so that participants could complete it while waiting for their doctor’s appointment, most constructs were assessed with one item measures, and we did not include validated questionnaires to assess food intake or physical activity. Lastly, the proportion of missing values was high for some variables, which is likely due to the pen-and-paper format, where participants could not be reminded of responding to all questions. Before using these variables in further analyses, users might want to test for systematic biases in missing responses.

Yet, by making this dataset available for further use, we hope to contribute to health psychology and behavioural medicine research and practice in two ways. First, this survey was used to inform the development of a multicomponent intervention that used digital elements such as a self-monitoring app and provided opportunities for exchange and social support between participants (Haderer et al., 2024). These are popular features of behavioural weight loss interventions, yet evidence for their effectiveness is somewhat mixed (Antoun et al., 2022). We thus hope that the data we collected provides helpful information for the development of new interventions. Second, the digital health divide had gained attention in research in recent years (König et al., 2023; Krukowski et al., 2024; Lyles et al., 2023), yet research on a potential rural-urban divide is scarce (Szinay et al., 2023). The preset dataset provides information on mobile health intervention use in a rural population and can also be used as a comparator to datasets collected primarily in urban populations. The data can also be used in meta-analyses to overcome the disadvantages of individual studies with small samples such as large confidence intervals, and so advance the study of the digital health divide.

## Supplementary Material

Supplementary material.docx

## Data Availability

Data, materials and analysis scripts are available on the Open Science Framework project page: https://osf.io/5vzpy/.

## Note

1 Per protocol, data collection was supposed to end on 20 January 2023, but due to changes in the project timeline, we were able to extend recruitment until February 2023.

## Open Scholarship

This article has earned the Center for Open Science badges for Open Data, Open Materials and Preregistered. The data and materials are openly accessible at https://osf.io/5vzpy/, https://osf.io/5vzpy/ and Methods: https://osf.io/2rngw; data analysis plan: https://osf.io/wdqvz.
