# Supplementary material for: Health behaviours and mobile intervention use in patients recruited from general practitioners’ practices in rural Bavaria
Source: Health Psychol Behav Med. 2024 Dec 23;13(1):2444244. doi: 10.1080/21642850.2024.2444244 (PMC11703449; doi:10.1080/21642850.2024.2444244)
Supplement: Supplementary material.docx [file RHPB_A_2444244_SM1349.docx]

# Supplementary material: Tables

Table S1. Assignments of highest school-leaving and vocational qualifications to years of education. Years of education were calculated as the sum of the two scores.

| Questionnaire item (German) | English translation | Code assigned in questionnaire | Recoded into years |
| --- | --- | --- | --- |
| **Highest school-leaving qualification** | | | |
| habe (noch) keinen Schulabschluss | Do not have a school leaving certificate (yet) | 1 | 8 |
| Volksschl-, Hauptschulabschluss | Elementary school, lower secondary school | 2 | 9 |
| Mittlere Reife, Realschulabschluss | Intermediate secondary school leaving certificate | 3 | 10 |
| Abschluss der Polytechnischen Oberschule | Completion of polytechnic secondary school | 4 | 11 |
| Abschluss einer (Berufs-)Fachschule | Graduation from a (vocational) technical school | 5 | 12 |
| Abitur | University entrance qualification (Abitur) | 6 | 13 |
| anderer Abschluss, und zwar: | Other qualification, namely: ____________________ | 7 | 11 |
| **Highest vocational qualification** |  |  |  |
| habe (noch) keine abgeschlossene Berufsausbildung | Do not have completed vocational training (yet) | 1 | 0 |
| Berufsausbildung | Vocational training | 2 | 3 |
| Lehre | Apprenticeship | 3 | 3 |
| Meister | Master (craftsman) | 4 | 4.5 |
| Fachoberschule/ Berufsfachschule | Technical/vocational secondary school | 5 | 3 |
| Fachhochschule/ Hochschule | Degree from a university of applied science/degree from a university | 6 | 5 |
| Promotion/ Habilitation | PhD/doctorate/habilitation | 7 | 7 |
| anderer Abschluss, und zwar: | Other qualification, namely: ____________________ | 8 | 3 |

# Supplementary material: Summary of results for RQ 1

## Age

For fitness trackers and apps, a between-subjects ANOVA with the factor Stage revealed a significant main effect, *F*(4, 208) = 14.02, *p* < .001, *partial* η² = .21. According to Bonferroni-corrected post-hoc tests, unengaged participants (Stage 1) were significantly older than participants in all other stages (*p*s ≤ .027), while participants in the other stages did not differ significantly from one another.

For nutrition apps, the same between-subjects ANOVA also revealed a significant main effect of Stage, *F*(4,214) = 10.28, *p* < .001, *partial* η² = .16. Again, according to Bonferroni-corrected post-hoc tests, unengaged participants (Stage 1) were significantly older than participants in all others stages (*p*s ≤ .016), with the exception of them not differing significantly from participants who decided not to act (Stage 3; *p* = .101). Participants in all other stages did not differ significantly from one another.

Means and standard deviations for all Stages are presented in Table S2.

Table S2. Age and years of education by fitness tracker and app and nutrition app use stages (M (SD), n).

|  | Age | | Years of education | |
| --- | --- | --- | --- | --- |
|  | Fitness tracker and apps | Nutrition apps | Fitness tracker and apps | Nutrition apps |
| Stage 1 “unengaged” | 56.1 (14.1), 97 | 53.5 (14.7), 161 | 13.4 (2.3), 107 | 13.6 (2.6), 172 |
| Stage 2 “decided to act” | 44.1 (14.1), 15 | 40.0 (14.2), 14 | 12.7 (4.1), 16 | 13.1 (2.6), 15 |
| Stage 3 “decided not to act” | 32.8 (13.0), 11 | 41.0 (17.5), 10 | 14.1 (1.4), 11 | 13.1 (4.9), 10 |
| Stage 4 “acting” | 42.6 (14.1), 66 | 38.7 (17.6), 11 | 14.1 (3.0), 69 | 14.9 (3.8), 13 |
| Stage 5 “disengaged” | 44.3 (15.9), 24 | 37.8 (13.3), 23 | 14.3 (2.4), 25 | 14.3 (3.5), 23 |

## Education

For fitness trackers and apps, there was no significant main effect of Stage for years of education in a between-subjects ANOVA, *F*(4,223) = 1.51, *p* = .172, *partial* η² = .03. Similarly, there was no significant main effect of Stage for years of education in a between-subjects ANOVA for nutrition apps, *F*(4,228) = 1.14, *p* = .339, *partial* η² = .02.

## Gender

Since only one participant selected being of a diverse gender, this participant was excluded from the analysis. For fitness trackers and apps, a chi square test testing for gender differences in use stages was not statistically significant, χ²(*df* = 4) = 2.38, *p* = .666, Cramer’s V = .11. Similarly, the chi square test was not significant for nutrition apps, χ²(*df* = 4) = 3.80, *p* = .434, Cramer’s V = .13.

## Employment status

A chi square test looking at differences in fitness tracker and app use stages based on employment status was statistically significant, χ²(*df* = 20) = 61.75, *p* < .001, Cramer’s V = .26. Based on standardised residuals (± 1.96), it could be observed that participants with full-time employment used fitness trackers and apps (Stage 4) more frequently than expected, while they were less often unengaged non-users (Stage 1) than expected. Unemployed participants had more frequently decided to act (Stage 2) than expected. Participants who were still in education or training were less frequently unengaged (Stage 1) than expected but had more frequently decided not to act (Stage 3) or disengaged (Stage 5) from use. Finally, retired participants were more frequently unengaged (Stage 1) and less frequently actively using (Stage 4) than expected. These results, however, need to be interpreted with caution due to the small participant number per cell.

Also the chi square test for nutrition apps was statistically significant, χ²(*df* = 20) = 36.08, *p* = .015, Cramer’s V = .20. Participants in full-time employment used nutrition apps (Stage 4) more frequently than expected. Participants who were still in education or training were less frequently unengaged (Stage 1) and more frequently disengaged (Stage 5) from use than expected. Retired participants were more frequently unengaged (Stage 1) than expected.

## Migration history

Due to highly unequal sample sizes (*n*_migration history_ = 17 vs *n*_no migration history_ = 219), this analysis was not conducted.

## Income

Due to the large number of income brackets in the survey, the sample was divided into quartiles as outlined in Table S3^[[1]](#footnote-1)^. For fitness trackers and apps, the chi square test looking at differences in use stages based on income quartiles was not statistically significant, , χ²(*df* = 12) = 15.74, *p* = .203, Cramer’s V = .15. Also the chi square test for nutrition apps was not statistically significant, χ²(*df* = 12) = 6.66, *p* = .879, Cramer’s V = .10.

Table S3. Income quartiles

| Quartile number | Code(s) | Value(s) | % |
| --- | --- | --- | --- |
| 1 | 1-6 | “Less than 150€” – “1,500€ to 2,000€” | 36.5% |
| 2 | 7 | “2,000€ to 2,500€” | 16.0% |
| 3 | 8-9 | “2,500€ to 3,000€” – “3,000€ to 5,000€” | 34.2% |
| 4 | 10-11 | “5,000€ to 10,000€” – “more than 10,000€” | 13.3% |

2 In the preregistration, no binning was planned; due to the small sample size, reducing the number of categories was necessary to ensure sufficiently large sample sizes per cell.

1. In the preregistration, no binning was planned; due to the small sample size, reducing the number of categories was necessary to ensure sufficiently large sample sizes per cell. [↑](#footnote-ref-1)
